# The Clinical Course and Outcomes of Patients Hospitalized Due to COVID-19 during Three Pandemic Waves in Poland: A Single Center Observational Study

**DOI:** 10.3390/jcm11247386

**Published:** 2022-12-13

**Authors:** Carlo Bieńkowski, Justyna D. Kowalska, Marcin Paciorek, Piotr Wasilewski, Paweł Uliczny, Ewelina Garbacz-Łagożna, Andrzej Pihowicz, Monika Mrozińska, Tomasz Dyda, Michał Makowiecki, Joanna Puła, Andrzej Horban

**Affiliations:** 1 Hospital for Infectious Diseases in Warsaw, 01-201 Warsaw, Poland; 2 Department of Adults’ Infectious Diseases, Medical University of Warsaw, 01-201 Warsaw, Poland; 3 Faculty of Medicine, Collegium Medicum, Cardinal Stefan Wyszynski University in Warsaw, 01-201 Warsaw, Poland

**Keywords:** COVID-19, SARS-CoV-2, pandemic, epidemic waves

## Abstract

Background: The first case of coronavirus disease 2019 (COVID-19) in Poland was reported on 4 March 2020. We aim to compare the clinical course and outcomes of patients hospitalized in the Hospital for Infectious Diseases in Warsaw due to COVID-19 during three pandemic waves. Materials and methods: The medical data were collected for all patients diagnosed with COVID-19 hospitalized in our hospital from 6 March 2020 till 30 November 2021. COVID-19 diagnosis was confirmed by nasopharyngeal swabs using real-time polymerase chain reaction assay (RT-PCR) or SARS-CoV-2 antigen test. COVID-19 waves were defined based on the number and dynamics of cases. Results: Altogether, 2138 patient medical records were analyzed. The majority of the cohort was male (1235/2138, 57.8%), and the median age was 65 years [IQR: 50–74 years]. Patients hospitalized during the third wave had lower oxygen saturation on admission (*p* < 0.001) and were more likely to receive oxygen supplementation (*p* < 0.001). Serious complications, including pneumothorax (*p* < 0.001) and thromboembolic complications (*p* < 0.001), intensive care unit admission (*p* = 0.034), and death (*p* = 0.003), occurred more often in patients of the third wave. Conclusions: During the third wave, patients in our cohort experienced a more severe course of the disease and poorer outcomes.

## 1. Introduction and Background

At the end of 2019, a rapid increase in cases of severe acute respiratory syndrome, caused by the new coronavirus 2 (SARS-CoV-2) virus, was observed, and on 11 March, a new pandemic was declared [1]. In Poland, the first coronavirus disease 2019 (COVID-19) case was reported on 4 March 2020. Up until December 2022, in Poland, the incidence has been estimated at 0.94 per 100,000 population, with a total of 6,351,408 confirmed cases and 118,306 reported deaths [2].

Poland’s health policy has developed alongside the pandemic. However, at the beginning, every person with suspected SARS-CoV-2 infection had to be hospitalized, and every person with confirmed COVID-19 had to be isolated until the disease was no longer contagious. In addition, the testing policy was not unified, and not everyone was tested; therefore, some infections were not diagnosed. Testing for the variant types of SARS-CoV-2 was also not common practice [3]. However, we have data showing that different SARS-CoV-2 variants were causing more severe or less severe courses of the disease, with the delta variant (B.1.617.2) being the most dangerous and having the poorest outcomes [4,5,6].

Despite the introduction of immunomodulator drugs and antivirals, two and a half years after the pandemic started, an optimal treatment is still lacking. Data from real-world experience could be applicable to practice guidelines [7].

Vaccination against COVID-19 was introduced at the end of December 2020, but not everyone was eligible to receive the vaccine at that time. Healthcare workers were prioritized, and then elderly patients and those with underlying medical conditions [3,8].

The pandemic’s development changed during the different epidemic waves. An increase in the death rate of Polish citizens was observed throughout the whole pandemic and during each wave, which had a great influence on Polish society [9].

With multiple factors changing over time, we therefore aimed to investigate the clinical course and outcomes of patients hospitalized in a major infectious diseases hospital in Warsaw due to COVID-19 over three different pandemic waves. Despite the availability of national epidemiological data, it was important to characterize the data from a single center in which a standardized approach to the care of COVID-19 patients had been implemented.

## 2. Material and Methods

### 2.1. Local Standard of Care

Since March 2020, when the pandemic started in Poland, local standard operating procedures (SOPs) have been established in the Hospital for Infectious Diseases in Warsaw. These local SOPs unified questionnaires on medical history and standardized laboratory test panels on admission and during hospitalization, radiological diagnostics, and medical treatment, including etiotropic therapy, based on national guidelines [7,10,11], and were implemented into our standard of care. Our hospital’s bed capacity for COVID-19 patients was 96 beds across all wards and six beds for the intensive care units (ICUs).

Medical history comprised data on COVID-19 symptoms, including the onset of symptoms, concomitant diseases, and chronic treatment. In addition, every patient was assessed using the World Health Organization’s ordinal scale for clinical improvement on the day of admission [12].

The medical data for every patient diagnosed with COVID-19 who was hospitalized in the Hospital for Infectious Diseases in Warsaw from 6 March 2020 till 30 November 2021 were collected through a prospectively designed electronic case report form (eCRF). The data concerning laboratory tests performed during hospitalization were exported from the electronic database. Information on any treatment received during hospitalization, COVID-19 complications or other events, and outcomes were input into the eCRF by hospital physicians.

### 2.2. Study Design

Only patients diagnosed with SARS-CoV-2, which was based on positive results from nasopharyngeal swabs using real-time reverse transcriptase polymerase chain reaction assay (RT-PCR), or SARS-CoV-2 antigen test, were included in the analysis. Patients who were not infected, based on the tests, were not included in the analysis.

Nasopharyngeal swab samples collected from patients suspected of having a SARS-CoV-2 infection were stored for up to 48 h at 4–8 °C until they were analyzed in a dedicated viral transport inactivation and stabilization buffer. SARS-CoV-2 RNA from respiratory specimens was isolated using a ready set of IVD reagents based on the reverse magnetic bead capturing method: a TANBead Nucleic Acid Extraction Kit in combination with a Maelstrom 4800 automated nucleic acid purification platform (Taiwan Advanced Nanotech Inc., Taoyuan City, Taiwan). Qualitative testing for new coronavirus RNA was performed using a Viasure SARS-CoV-2 Real-Time PCR Detection IVD Kit (CerTest, San Mateo de Gallego, Zaragoza, Spain). The amplification and detection of fluorescence signals from specific molecular probes targeted at the ORF1ab (FAM channel) and N (ROX channel) genes of the SARS-CoV-2 gene sequences were completed using a Bio-Rad CFX96 thermocycler (Bio-Rad Laboratories, Inc., Hercules, CA, USA). The amplification parameters of the internal control were verified in terms of inhibition. The results were evaluated by laboratory personnel for the correct functioning of the process. An assessment of the results of the clinical sample tests was done after the examination and acceptance of valid positive and negative control results following each run. In accordance with the assay manufacturer’s recommendations, a Ct value of 40 was adopted as the cut-off value. In cases where SARS-CoV-2 target genes gave a negative result and there was an absence of signal, or that the Ct value was >40 of the internal control, the result was considered invalid, and retesting was requested.

COVID-19 waves were defined based on the number and the dynamics of the cases. A wave was distinguished, in Poland, after observing an increase, then a peak, followed by a decrease in new cases. In addition, these waves were identified by comparing the COVID-19 incidence increases and decreases with the SARS-CoV-2 variant that was dominant at that time [9,10,11,12,13]. We therefore defined the following three COVID-19 waves: the first wave from 6 March 2020 to 31 January 2021, the second wave from 1 February 2021 to 31 July 2021, and the third wave from 1 August 2021 to 30 November 2021.

Only variables that were available for 85% or more of the patients were included in the analysis.

### 2.3. Statistical Analysis

Non-parametric tests were used for group comparisons, the Kruskal–Wallis test for continuous variables, and the Chi-squared test for nominal variables. A value of *p* < 0.05 was considered significant, and all statistical tests were two-sided.

Logistic regression models were used to identify factors associated with death for each wave separately. Candidate predictors were entered into the model irrespective of the results of the univariate analysis. After entering all variables into the model, the variables that showed the least significant associations were subsequently excluded until all variables remained significant (*p* < 0.05).

Factors that were significant in the univariate models (*p* < 0.0.5) were included in the multivariate model and are presented as odds ratios (OR) at 95% CI (confidence intervals).

The study was conducted according to the *Strengthening the Reporting of Observational Studies in Epidemiology* (STROBE) guidelines [13].

Statistical analyses were performed using the R program, version 4.1.1 (2021, Vienna, Austria).

## 3. Results

In total, the medical records of 2138 patients were analyzed. The median age of our patients was 65 years [IQR: 50–74 years]. The most common age group was for patients aged >70 years (743/2138; 34.8%). The majority of the cohort consisted of male patients (1235/2138; 57.8%). Healthcare workers constituted 6.5% of all our patients (132/2138). On admission 1377/2138 (64.4%) of patients required oxygen supplementation, and presented with a median oxygen saturation of 93% [IQR: 88%–97%]. The three most common COVID-19 symptoms were cough (1655/2138; 79.4%), malaise (1557/2138; 74.8%), and fever (1560/2138; 74.6%). After one week of hospitalization, 864/2138 (40.4%) people required oxygen therapy. The two most common complications were superinfections, including nosocomial infections (261/2138; 12.3%), and bacterial pneumonia (248/2138; 11.7%). Unfavorable COVID-19 outcomes were defined as ICU admission or death, and these occurred in 180/2138 (8.4%) and 274/2138 (12.8%) cases, respectively (See Table 1).

### 3.1. Comparison between Three Pandemic Waves

#### 3.1.1. Clinical Evaluation on Admission and Comorbidities

We compared patients’ characteristics and clinical outcomes across three time periods. The majority of hospitalized patients were aged >70 years for all three waves (1st, 430/1225, 35.1%; 2nd, 235/687, 34.2%; 3rd, 78/226, 34.5%; *p* = 0.008). Healthcare workers were more likely to be hospitalized during the first pandemic wave than the second and third waves (112/1225, 9.7% vs. 14/687, 2.1% vs. 6/226, 2.7%, respectively. *p* > 0.001). In terms of comorbidities, having a history of myocardial infarction was more frequent in patients during the second wave than the first and third waves (70/687, 10.3% vs. 65/1225, 5.4% vs. 17/226, 7.6%, respectively. *p* < 0.001). Diabetes mellitus requiring pharmacotherapy, but without diabetes-associated organ damage, was also more often found in patients of the second wave than in those patients hospitalized during the first and third waves (135/687, 19.7% vs. 176/1225, 14.4% vs. 28/226, 12.5%, respectively. *p* = 0.016). Any kidney failure defined as having a glomerular filtration rate <60 mL/min/1.73 m^2^ at admission was more common in third-wave patients compared to those hospitalized during the first and second waves (50/226, 22.1% vs. 186/1225, 15.3% vs. 139/687, 20.6%, respectively. *p* = 0.003). Patients who had abused alcohol up to 30 days before hospital admission were more likely to be hospitalized during the third compared to the first and second waves (10/226, 5.0% vs. 27/1225, 2.3% vs. 26/687, 4.2%, respectively. *p* = 0.032) (Table 1). Patients hospitalized during the first wave had higher oxygen saturation on admission than those hospitalized during the second and third waves (median 94% [IQR: 90–97%] vs. median 90% [IQR: 85–94%] vs. median 91% [IQR: 86.5–96%], respectively. *p* < 0.001). During the second wave, more patients required oxygen supplementation (537/687, 78.2% vs. 679/1225, 55.3% vs. 162/226, 71.7%. *p* < 0.001) than first- and third-wave hospitalized patients, respectively (Table 1). The shortest time interval between the first symptoms and hospital admission was observed in patients hospitalized during the third wave compared to those admitted during the first and second waves (median 7 days [IQR 5–10] vs. median 8 days [IQR: 6–11] vs. median 8 days [IQR: 6–11], respectively. *p* = 0.022). The three most common COVID-19 symptoms were more likely to be present in patients hospitalized during the second wave compared to those admitted during the first and third waves (658/687, 98.2% vs. 1136/1225, 93.3% vs. 218/226, 96.9%, respectively. *p* < 0.001). However, symptoms such as musculoskeletal pain (497/1225, 42.1% vs. 272/687, 41.3% vs. 67/226, 31.2%. *p* = 0.010), chest pain (229/1225, 19.4% vs. 98/687, 14.9% vs. 24/226, 11.2%. *p* = 0.003), dysgeusia (300/1225, 25.4% vs. 131/687, 19.9% vs. 33/226, 15.6%. *p* < 0.001), dysosmia (299/1225, 25.3% vs. 118/687, 17.9% vs. 35/226, 16.5%. *p* < 0.001), and headache (363/1225, 30.7% vs. 181/687, 27.5% vs. 46/226, 21.9%. *p* = 0.024) were more frequently present in patients during the first COVID-19 wave than the second and third waves, respectively. On the other hand, malaise (543/687, 81.7% vs. 852/1225, 71.1% vs. 162/226, 74.2%. *p* < 0.001) and dyspnea (465/687, 69.7% vs. 690/1225, 57.3% vs. 146/226, 65.8%. *p* < 0.001) were more frequent in patients during the second wave compared to those hospitalized during the first and third pandemic waves, respectively (Table 1).

#### 3.1.2. Laboratory Findings

In terms of baseline laboratory measurement results, the patients hospitalized during the second pandemic wave had higher inflammatory markers (C-reactive protein concentration in mg/L: 69.0 [IQR: 43.8–166.0] vs. 60.0 [IQR: 30.0–159.2] vs. 63.5 [IQR: 32.5–148.0]. *p* < 0.001; procalcitonin concentration in ng/mL: 0.1 [IQR: 0.0–0.2] vs. 0.0 [0.0–0.1] vs. 0.1 [IQR: 0.1–0.4]. *p* < 0.001; and interleukin 6 concentration in pg/mL: 51.8 [IQR: 22.4–95.1] vs. 37.9 [IQR: 16.1–77.4] vs. 40.2 [IQR: 16.1–100.0]. *p* < 0.001); higher D-dimers concentration in ng/L (1127.6 [IQR: 775.1–1818.3] vs. 1011.8 [IQR: 638.0–1793.3] vs. 1076.4 [625.0–1781.5]. *p* = 0.002); and higher fibrinogen concentration in g/L (7.0 [IQR: 5.6–8.7] vs. 6.5 [5.2–8.1] vs. 6.1 [4.7–7.9]. *p* < 0.001) compared to patients admitted during the first and third waves, respectively.

#### 3.1.3. Treatment

We also compared the use of specific treatments across the three waves. Patients during the first wave were more likely to be treated with chloroquine (1st, 26/1225, 2.1% vs. 2nd, 1/687, 0.1% vs. 3rd, 1/226, 0.4%. *p* = 0.001), hydroxychloroquine (32/1225, 2.7% vs. 0/687, 0.0% vs. 0/226, 0.0%, respectively. *p* < 0.001), and azithromycin (259/1225, 22.1% vs. 98/687, 15.0% vs. 17/226, 7.7%, respectively. *p* < 0.001) compared to the remaining waves. However, patients hospitalized during the second wave were more frequently treated with remdesivir (2nd, 466/786, 68.6% vs. 1st, 434/1225, 35.8% vs. 3rd, 135/226, 60.5%. *p* < 0.001), steroids (547/687, 80.7% vs. 639/1225, 52.8% vs. 165/226, 73.0%, respectively. *p* < 0.001), heparin (627/687, 94.4% vs. 914/1225, 77.1% vs. 196/226, 87.5%, respectively. *p* < 0.001), and other antibiotics than azithromycin (589/687, 88.3% vs. 888/1225, 74.4%, vs. 169/226, 76.1%, respectively. *p* < 0.001). At the same time, tocilizumab was more frequently administered to patients hospitalized during the third wave (9/226, 4.0% vs. 16/1225, 1.3% vs. 11/687, 1.6%. *p* = 0.015) (Table 1) than the first and third, respectively.

#### 3.1.4. Complications

Serious complications including pneumothorax (10/226, 4.5% vs. 5/1225, 0.4% vs. 11/687, 1.6%. *p* < 0.001) and thromboembolic complications (26/226, 11.6% vs. 46/1225, 3.8% vs. 57/687, 8.4%. *p* < 0.001), intensive care unit admission (28/226, 12.4% vs. 90/1225, 7.3% vs. 62/687, 9.0%. *p* = 0.034), and death (36/226, 16.8% vs. 131/1225, 10.7% vs. 105/687, 15.3%. *p* = 0.003) were more often in patients hospitalized during the third pandemic wave compared to those admitted during the first and second wave, respectively. However, ventricular arrhythmias were more frequent in patients during the second wave compared to those admitted during the first and third waves (16/687, 2.4% vs. 7/1225 vs. 2/226, 0.9%, respectively. *p* = 0.002) (Table 1).

#### 3.1.5. Multivariate Analysis

**Figure 1:**
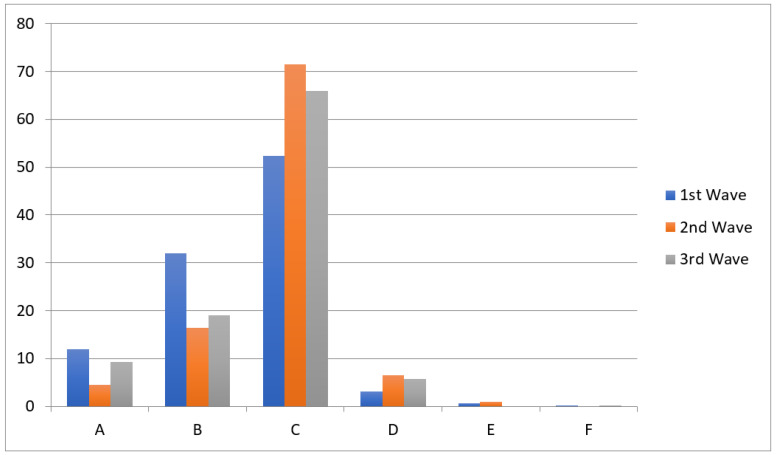
Ordinal scale for clinical improvement on the day of admission for patients hospitalized in the Hospital for Infectious Diseases in Warsaw due to COVID-19 between March 2020 and November 2021 (*p* < 0.001). Results are presented in percentage (%) of patients (*y*-axis). Legend: (A) Hospitalized, not requiring oxygen supplementation and not requiring medical care. (B) Hospitalized, not requiring oxygen supplementation but requiring medical care. (C) Hospitalized, requiring normal oxygen supplementation. (D) Hospitalized, requiring non-invasive ventilation with high-flow oxygen equipment (helmet, high-flow oxygen nasal cannula, HFNC). (E) Hospitalized, requiring invasive mechanical ventilation or ECMO. (F) Death.

**Figure 2:**
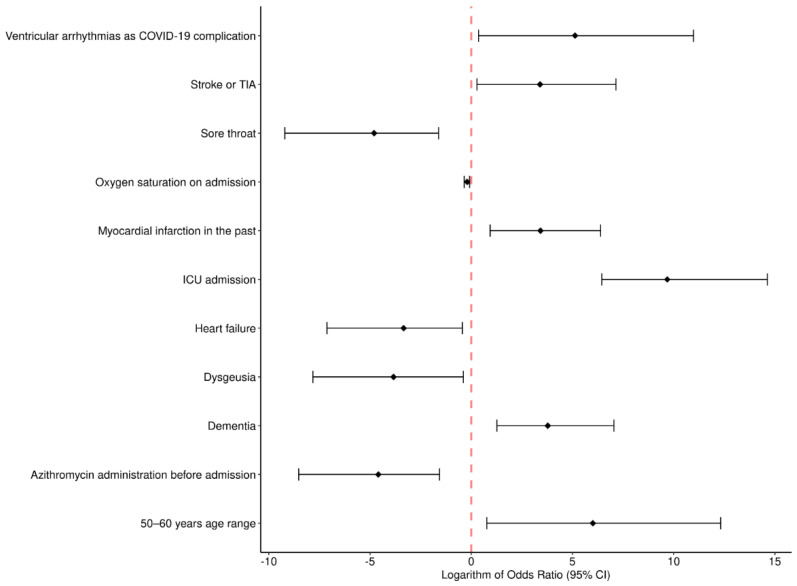
Multivariate logistic regression model analysis of the factors independently associated with death during the first COVID-19 wave in the Hospital for Infectious Diseases in Warsaw (Poland).

Logistic regression model analysis showed the factors that were independently associated with death in COVID-19 patients hospitalized during the three pandemic waves in the hospital (Figure 1 and Figure 2).

**Figure 3:**
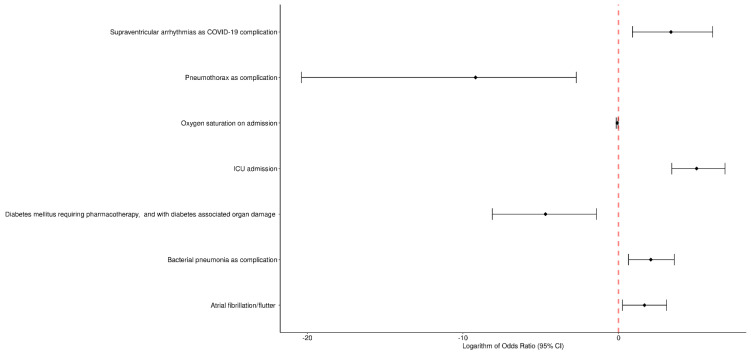
Multivariate logistic regression model analysis of the factors independently associated with death during the second COVID-19 wave in the Hospital for Infectious Diseases in Warsaw (Poland).

1. During the first wave (Figure 2):
  - 50–60 years age range, OR 407.37; 95% CI 2.17–223,060.46, *p* = 0.035;
  - Oxygen saturation on admission, OR 0.82; 95% CI 0.71–0.92, *p* = 0.003;
  - Myocardial infarction in the past, OR 30.44; 95% CI 2.53–597.84, *p* = 0.011;
  - Heart failure, OR 0.04; 95% CI 0.00–0.65, *p* = 0.042;
  - Stroke or TIA, OR 29.86; 95% CI 1.33–1278.97, *p* = 0.047;
  - Dementia, OR 43.93; 95% CI 3.54–1158.45, *p* = 0.008;
  - Sore throat, OR 0.01; 95% CI 0.00–0.20, *p* = 0.012;
  - Dysgeusia, OR 0.02; 95% CI 0.00–0.68, *p* = 0.041;
  - Ventricular arrhythmias as COVID-19 complication, OR 168.58; 95% CI 1.43–56,448.69, *p* = 0.045;
  - ICU admission, OR 15,973.93; 95% CI 634.60–2,260,123.88, *p* < 0.001;
  - Azithromycin administration before admission, OR 0.01; 95% CI 0.00–0.21, *p* = 0.010.
2. During the second wave (Figure 3):
  - Oxygen saturation on admission, OR 0.92; 95% CI 0.86–0.98, *p* = 0.008;
  - Diabetes mellitus requiring pharmacotherapy, and with diabetes-associated organ damage, OR 0.01; 95% CI 0.00–0.24, *p* = 0.006;
  - Atrial fibrillation/flutter, OR 5.28; 95% CI 1.29–22.09, *p* = 0.020;
  - Supraventricular arrhythmias as COVID-19 complication, OR 29.09; 95% CI 2.46–426.20, *p* = 0.010;
  - Pneumothorax as complication, OR <0.001; 95% CI 0.00–0.07, *p* = 0.014;
  - Bacterial pneumonia as complication OR, 7.98; 95% CI 1.90–35.79, *p* = 0.005;
  - ICU admission, OR 151.44; 95% CI 30.98–943.98, *p* < 0.001.
3. During the third wave:
  - None of the factors that were significant in the univariate model were significant in the multivariate analysis.

## 4. Discussion

To our knowledge, this is the first study in Poland conducted in a single center that compares the clinical features and outcomes of patients hospitalized due to COVID-19 during three pandemic waves. Other studies from around the world comparing COVID-19 waves are difficult to compare with our study due to the different wave definitions and the different SARS-CoV-2 variants dominating each wave [14,15,16,17].

However, in Poland, there have been studies where death-associated factors were analyzed, but only in comparison to two pandemic waves or to one [18,19]. In addition, it was important to characterize the data from a single center in which a standardized approach to COVID-19 patient care had been implemented. Every piece of real-life data might be useful for the management of future COVID-19 patients.

COVID-19 has mainly been asymptomatic or taken a mild course; however, the clinical spectrum of the disease is vast and includes severe progressive pneumonia and acute respiratory distress syndrome, both of which may be accompanied by a cytokine storm, thromboembolic complications, and/or multiple organ dysfunction [1,20,21]. Regarding the fact that patients with more severe courses require hospitalization, our cohort consisted of individuals who were most vulnerable to infection; therefore, the most numerous group were elderly patients. In addition, these patients more often had underlying medical conditions that may have predisposed them to COVID-19 and required medical care. However, during the first pandemic wave, every patient with a suspected SARS-CoV-2 infection had to undergo hospital observation; and so, during the first wave, a milder course may have been observed (Table 1).

The clinical evaluation on admission revealed that patients hospitalized during the first wave were less severely hypoxic. However, they had a more diverse range of different symptoms. During this period, the alfa variant was the most commonly observed variant in our region [2,22,23,24]. On the other hand, the shortest period from disease onset to hospital admission was observed in the third wave. Moreover, inflammatory markers were also more elevated in patients during this period, which may correspond to the fact that, during this wave, the delta variant was the most dominant form in our region [2,22,23,24]. 

During the pandemic, a search began for a safe and effective COVID-19 treatment [25,26]. At the beginning, there were some data suggesting that chloroquine and hydroxychloroquine (anti-inflammatory drugs) may have reduced the mortality rate in SARS-CoV-2 infected individuals, especially when the therapy was combined with azithromycin. However, the meta-analyses show otherwise; what is more, this combination of drugs may have increased mortality [27,28,29]. Macrolides themselves also did not show any beneficial effect for patients with COVID-19 [30]. It was the case that these drugs were used significantly more frequently in our hospital during the first pandemic wave, when there was still not much data on the treatment’s efficacy and safety, although we did not observe a higher mortality in our cohort during this period (Table 1).

During the second wave, more valuable data were obtained, and treatment recommendations were more certain [31]. Therefore, in our cohort during this period, we observed significantly increased administration of remdesivir and corticosteroids (Table 1). Moreover, due to new and improved data on bacterial superinfections and thromboembolic complications, a significant increase in antibiotics other than both azithromycin and heparin administration was observed (Table 1).

As for tocilizumab, there were some conflicting reports, some suggesting that it does not improve clinical presentation when combined with standards of care [32]. However, Flisiak et al. showed that tocilizumab administration in patients with SARS-CoV-2 infection reduces mortality and speeds up clinical improvement if the patient has a high IL-6 concentration and requires oxygen supplementation [33]. These data were published in the middle of the second pandemic wave in Poland; therefore, its implementation in everyday treatment was observed to be significantly higher during the third wave (Table 1).

In Poland, vaccines for COVID-19 were introduced at the end of December 2020 [8]. The fact that healthcare workers (HCWs) are at risk of acquiring the infection during their work duties may explain why the biggest number of HCWs were hospitalized due to COVID-19 during the first wave, when the vaccines had not yet been introduced [8,34].

Severe complications, including ICU admission and death, were most common during the third wave, which also corresponded to disease severity on admission and the delta variant being dominant [2,22,23,24,35].

We have shown many factors that influenced deaths during the first and second pandemic waves; however, none of these seemed to be death predictors in the third pandemic wave, when our patients had the worst outcomes. Our study has more of a descriptive nature, and we have aimed to show medical history data, comorbidities, treatment, and COVID-19 complications.

Some important limitations are evident due to the fact that our study is of a retrospective, observational nature. First, most of our patients were symptomatic, as asymptomatic cases were less likely to seek medical care. However, during the beginning of the first wave, every patient suspected of being SARS-CoV-2 infected was hospitalized, which may provide some clinical presentation bias when being compared to the second and third waves. Moreover, the clinical course of the disease may have been influenced by the currently applied standard of care, which varied over time as new recommendations were introduced. Moreover, due to the large number of patients in each population, it is easier to obtain significant results in the univariate analysis.

There are also some strengths worth mentioning however: we had a large, representative cohort of 2138 COVID-19 patients with known outcomes. Moreover, we had local SOPs for collecting medical history, laboratory testing, and patient management, which altogether provided a more unified system for the management of patients with COVID-19.

To conclude; we have described patients’ characteristics at baseline for three pandemic waves. We also found that there were some differences between the waves in comorbidities, treatment, and complications. The data indicates that for all three waves, COVID-19 was a severe disease in hospitalized patients with a high risk of poor outcomes. The patients of the third wave were the most severely ill on admission and had poorer outcomes; however, none of the factors influencing death during the first and second wave predicted death in the third wave.

## Figures and Tables

**Table 1:** Baseline characteristics and clinical data for patients hospitalized in the Hospital for Infectious Diseases in Warsaw due to COVID-19 during three pandemic waves from 2020 to 2021 in Poland.

Characteristic	Total	1st Wave	2nd Wave	3rd Wave	*p*-Value
	*n* = 2138	*n* = 1225	*n* = 687	*n* = 226	
Age in years, median [IQR *]	64 [50–74]	63 [48–73]	65 [53–74]	64 [50–74]	0.051
Aged 18–50 years, *n* (%)	527 (24.6)	323 (26.4)	145 (21.1)	59 (26.1)	0.008
Aged 50–60 years, *n* (%)	326 (15.2)	190 (15.5)	97 (14.1)	39 (17.3)	
Aged 60–70 years, *n* (%)	542 (25.4)	282 (23.0)	210 (30.6)	50 (22.1)	
Aged >70 years, *n* (%)	743 (34.8)	430 (35.1)	235 (34.2)	78 (34.5)	
Male sex, *n* (%)	1235 (57.8)	726 (59.3)	380 (55.3)	129 (57.1)	0.238
BMI in kg/m^2^, median [IQR]	28.4 [25.2–32.4]	28.3 [24.9–32.0]	28.5 [26.0–32.8]	29.0 [24.6–33.8]	0.08
Healthcare worker, *n* (%)	132 (6.5)	112 (9.7)	14 (2.1)	6 (2.7)	<0.001
**Comorbidities**					
Past myocardial infarction, *n* (%)	152 (7.2)	65 (5.4)	70 (10.3)	17 (7.6)	<0.001
Heart failure, *n* (%)	229 (10.8)	131 (10.8)	71 (10.4)	27 (12.1)	0.781
Atrial fibrillation/flutter, *n* (%)	191 (9.1)	113 (9.3)	61 (9.0)	17 (7.6)	0.701
Hypertension, *n* (%)	1042 (49.1)	588 (48.4)	341 (50.0)	113 (50.4)	0.732
Peripheral vascular disease, *n* (%)	73 (3.5)	39 (3.2)	31 (4.6)	3 (1.4)	0.062
Stroke or TIA **, *n* (%)	94 (4.4)	49 (4.0)	36 (5.3)	9 (4.0)	0.439
Hemiplegia, *n* (%)	65 (3.1)	33 (2.7)	27 (4.0)	5 (2.3)	0.244
Dementia, *n* (%)	111 (5.2)	59 (4.9)	33 (4.8)	19 (8.4)	0.072
COPD ***, *n* (%)	111 (5.2)	59 (4.9)	37 (5.4)	15 (6.8)	0.486
Asthma, *n* (%)	134 (6.4)	79 (6.6)	47 (6.9)	8 (3.6)	0.197
Interstitial lung disease, *n* (%)	20 (0.9)	9 (0.7)	8 (1.2)	3 (1.4)	0.524
Connective tissue disease, *n* (%)	45 (2.1)	24 (2.0)	15 (2.2)	6 (2.7)	0.784
Gastric ulcer, *n* (%)	60 (2.8)	39 (3.2)	18 (2.6)	3 (1.3)	0.28
Liver disease					
None, *n* (%)	2068 (97.6)	1177 (97.1)	674 (98.7)	217 (97.2)	0.111
Chronic hepatitis or cirrhosis without portal hypertension, *n* (%)	39 (1.8)	24 (2.0)	9 (1.3)	6 (2.7)	
Cirrhosis and portal hypertension with no history of esophageal varices bleeding, *n* (%)	10 (0.5)	10 (0.8)	0 (0.0)	0 (0.0)	
Cirrhosis and portal hypertension with a history of bleeding from esophageal varices, *n* (%)	1 (0.0)	1 (0.1)	0 (0.0)	0 (0.0)	
Diabetes mellitus					
None or diet-controlled, *n* (%)	1725 (81.1)	1008 (82.8)	530 (77.5)	187 (83.5)	0.016
Requiring pharmacotherapy, without diabetes-associated organ damage, *n* (%)	339 (15.9)	176 (14.4)	135 (19.7)	28 (12.5)	
Requiring pharmacotherapy, with diabetes-associated organ damage, *n* (%)	62 (2.9)	34 (2.8)	19 (2.8)	9 (4.0)	
Kidney failure ****, *n* (%)	375 (17.7)	186 (15.3)	139 (20.6)	50 (22.1)	0.003
Tumor					
None, *n* (%)	1988 (93.9)	1150 (94.6)	631 (92.9)	207 (92.8)	0.586
Without metastases, *n* (%)	108 (5.1)	54 (4.4)	40 (5.9)	14 (6.3)	
With metastases, *n* (%)	22 (1.0)	12 (1.0)	8 (1.2)	2 (0.9)	
Lymphoma, *n* (%)	26 (1.2)	15 (1.2)	7 (1.0)	4 (1.8)	0.666
AIDS *****, *n* (%)	24 (1.1)	15 (1.2)	8 (1.2)	1 (0.5)	0.596
HIV ****** infection					
None, *n* (%)	2090 (98.7)	1199 (98.6)	668 (98.5)	223 (99.6)	0.801
Not treated	6 (0.3)	4 (0.3)	2 (0.3)	0 (0.0)	
On treatment	22 (1.0)	13 (1.1)	8 (1.2)	1 (0.4)	
Immunosuppressive treatment, *n* (%)	61 (2.9)	35 (2.9)	16 (2.4)	10 (4.5)	0.262
Past alcohol abuse (>1 month), *n* (%)	63 (3.2)	27 (2.3)	26 (4.2)	10 (5.0)	0.032
Alcohol abuse during last month, *n* (%)	37 (2.0)	19 (1.8)	11 (1.8)	7 (3.4)	0.272
Smoker, *n* (%)	116 (5.9)	63 (5.4)	40 (6.5)	13 (6.4)	0.625
Non-smoker for at least for 6 months, *n* (%)	336 (17.4)	181 (16.0)	121 (20.0)	34 (17.2)	0.113
**Clinical evaluation on admission**					
Oxygen saturation on admission as %, median [IQR]	93.0 [88.0–97.0]	94.0 [90.0–97.0]	90.0 [85.0–94.0]	91.0 [86.5–96.0]	<0.001
Time interval between first symptoms and admission in days, median [IQR]	8.0 [6.0–11.0]	8.0 [6.0–11.0]	8.0 [6.0–11.0]	7.0 [5.0–10.0]	0.022
The three most common symptoms (malaise, fever, cough), *n* (%)	2012 (95.3)	1136 (93.3)	658 (98.2)	218 (96.9)	<0.001
Fever >38 degrees Celsius, *n* (%)	1560 (74.6)	880 (73.0)	513 (77.6)	167 (74.9)	0.088
Musculoskeletal pain, *n* (%)	836 (40.7)	497 (42.1)	272 (41.3)	67 (31.2)	0.01
Sore throat, *n* (%)	306 (14.9)	170 (14.4)	106 (16.1)	30 (14.2)	0.579
Rhinitis, *n* (%)	256 (12.5)	135 (11.5)	86 (13.1)	35 (16.7)	0.094
Cough, *n* (%)	1655 (79.4)	934 (77.7)	544 (81.9)	177 (81.2)	0.077
Dyspnea, *n* (%)	1301 (62.2)	690 (57.3)	465 (69.7)	146 (65.8)	<0.001
Chest pain, *n* (%)	351 (17.1)	229 (19.4)	98 (14.9)	24 (11.2)	0.003
Hemoptysis, *n* (%)	51 (2.5)	25 (2.1)	19 (2.9)	7 (3.3)	0.435
Dysgeusia, *n* (%)	464 (22.6)	300 (25.4)	131 (19.9)	33 (15.6)	<0.001
Dysosmia, *n* (%)	452 (22.0)	299 (25.3)	118 (17.9)	35 (16.5)	<0.001
Headache, *n* (%)	590 (28.8)	363 (30.7)	181 (27.5)	46 (21.9)	0.024
Nausea/emesis, *n* (%)	369 (18.0)	209 (17.7)	132 (20.1)	28 (13.1)	0.062
Diarrhea, *n* (%)	488 (23.7)	263 (22.2)	177 (26.9)	48 (22.2)	0.07
Abdominal pain, *n* (%)	191 (9.3)	116 (9.8)	65 (9.9)	10 (4.7)	0.052
Malaise, *n* (%)	1557 (74.8)	852 (71.1)	543 (81.7)	162 (74.3)	<0.001
Conjunctivitis, *n* (%)	66 (3.2)	34 (2.9)	29 (4.4)	3 (1.4)	0.059
**Laboratory findings on admission**					
C-reactive protein concentration in mg/L (norm: <10 mg/L), median [IQR]	63.0 [34.0–160.0]	60.0 [30.0–159.2]	69 [43.8–166.0]	63.5 [32.5–148.0]	<0.001
Procalcitonin concentration in ng/ml (norm: <0.5 ng/ml), median [IQR]	0.1 [0.0–0.2]	0.0 [0.0–0.1]	0.1 [0.0–0.2]	0.1 [0.1–0.4]	<0.001
Interleukin 6 concentration in pg/ml (norm: <6.65 pg/ml), median [IQR]	43.2 [18.2–87.3]	37.9 [16.1–77.4]	51.8 [22.4–95.1]	40.2 [16.1–100.0]	<0.001
D-dimers concentration in ng/L (norm: <500 ng/L), median [IQR]	1059.8 [680.2–1799.0]	1011.8 [638.0–1793.3]	1127.6 [775.1–1818.3]	1076.4 [625.0–1781.5]	0.002
Fibrinogen concentration in g/L (norm: 2.2–5.0), median [IQR]	6.7 [5.3–8.2]	6.5 [5.2–8.1]	7.0 [5.6–8.7]	6.1 [4.7–7.9]	<0.001
Platelet count in 1000 cells/mm^3^ (norm: 125.3–396.2 cells/mm^3^), median [IQR]	214.0 [163.0–282.0]	219.0 [168.0–291.0]	207.0 [157.2–271.0]	204.0 [156.2–271.5]	0.001
Creatinine concentration between 46 and 92 μmol/L, *n* (%)	1477 (69.9)	868 (71.6)	467 (69.2)	142 (62.8)	0.101
Creatinine concentration <46 μmol/L, *n* (%)	81 (3.8)	46 (3.8)	26 (3.9)	9 (4.0)	
Creatinine concentration >92 μmol/L, *n* (%)	555 (26.3)	298 (24.6)	182 (27.0)	75 (33.2)	
Urea concentration between 2.5 and 7.1 mmol/L, *n* (%),	1350 (64.0)	794 (65.6)	427 (63.5)	129 (57.1)	0.105
Urea concentration >7.1 mmol/L, *n* (%)	702 (33.3)	388 (32.1)	225 (33.5)	89 (39.4)	
Sodium concentration between 137 and 145 mmol/L, *n* (%)	1229 (58.4)	739 (61.2)	344 (51.0)	146 (65.2)	<0.001
Sodium concentration <137 mmol/L, *n* (%)	830 (39.4)	447 (37.0)	317 (47.0)	66 (29.5)	
Sodium concentration >145 mmol/L, *n* (%)	47 (2.2)	22 (1.8)	13 (1.9)	12 (5.4)	
Potassium concentration between 3.6 and 5.0 mmol/L, *n* (%)	1704 (80.9)	984 (81.4)	538 (79.8)	182 (81.2)	0.421
Potassium concentration <3.6 mmol/L, *n* (%)	299 (14.2)	166 (13.7)	106 (15.7)	27 (12.1)	
Potassium concentration >5.0 mmol/L, *n* (%)	59 (4.9)	30 (4.5)	15 (6.7)	104 (4.9)	
Alanine aminotransferase activity between 4 and 35 U/L in women and 4 and 50 U/L in men, *n* (%)	1327 (63.0)	804 (66.3)	394 (58.7)	129 (57.3)	<0.001
Alanine aminotransferase concentration >35 U/L in women and >50 U/L in men, *n* (%)	781 (37.0)	408 (33.7)	277 (41.3)	96 (42.7)	
Aspartate aminotransferase activity between 10 and 36 U/L in women and 10–59 U/L in men, *n* (%)	1034 (49.2)	659 (54.6)	278 (41.7)	97 (42.9)	<0.001
Aspartate aminotransferase >36 U/L in women and >59 U/L in men, *n* (%)	1066 (50.8)	548 (45.4)	389 (58.3)	129 (57.1)	
Lipase activity in U/L between 23 and 300 U/L, *n* (%)	1602 (82.7)	883 (82.6)	533 (81.7)	186 (86.1)	0.34
Lipase activity >300 U/L, *n* (%)	304 (15.7)	172 (16.1)	107 (16.4)	25 (11.6)	
Phosphocreatine kinase between 30 and 135 U/L in women and 30 and 170 U/L in men, *n* (%)	855 (42.3)	511 (44.3)	266 (40.7)	78 (36.1)	<0.001
Phosphocreatine kinase >135 U/L in women and >170 U/L in men, *n* (%)	712 (35.2)	356 (30.9)	257 (39.3)	99 (45.8)	
**Treatment**					
Chloroquine, *n* (%)	28 (1.3)	26 (2.1)	1 (0.1)	1 (0.4)	0.001
Hydroxychloroquine, *n* (%)	32 (1.5)	32 (2.7)	0 (0.0)	0 (0.0)	<0.001
Tocilizumab, *n* (%)	36 (1.7)	16 (1.3)	11 (1.6)	9 (4.0)	0.015
Remdesivir, *n* (%)	1035 (49.0)	434 (35.8)	466 (68.6)	135 (60.5)	<0.001
Steroids, *n* (%)	1351 (63.9)	639 (52.8)	547 (80.7)	165 (73.0)	<0.001
Heparin, *n* (%)	1737 (83.8)	914 (77.1)	627 (94.4)	196 (87.5)	<0.001
Azithromycin, *n* (%)	374 (18.3)	259 (22.1)	98 (15.0)	17 (7.7)	<0.001
Other antibiotics, *n* (%)	1646 (79.0)	888 (74.4)	589 (88.3)	169 (76.1)	<0.001
**Complications during hospitalization**					
Supraventricular arrhythmias, *n* (%)	52 (2.5)	23 (1.9)	22 (3.3)	7 (3.2)	0.144
Ventricular arrhythmias, *n* (%)	25 (1.2)	7 (0.6)	16 (2.4)	2 (0.9)	0.002
Myocardial infarction, *n* (%)	37 (1.7)	15 (1.2)	17 (2.5)	5 (2.2)	0.114
Stroke, *n* (%)	16 (0.8)	6 (0.5)	9 (1.3)	1 (0.4)	0.118
Pneumothorax, *n* (%)	26 (1.2)	5 (0.4)	11 (1.6)	10 (4.5)	<0.001
Nosocomial infection, *n* (%)	261 (12.3)	133 (11.0)	100 (14.7)	28 (12.5)	0.062
Bacterial pneumonia, *n* (%)	248 (11.7)	129 (10.7)	90 (13.2)	29 (12.9)	0.209
Thromboembolic complications, *n* (%)	129 (6.1)	46 (3.8)	57 (8.4)	26 (11.6)	<0.001
Gastrointestinal hemorrhage, *n* (%)	20 (0.9)	16 (1.3)	3 (0.4)	1 (0.4)	0.119
ICU ****** admission, *n* (%)	180 (8.4)	90 (7.3)	62 (9.0)	28 (12.4)	0.034
Death, *n* (%)	274 (12.8)	131 (10.7)	105 (15.3)	36 (16.8)	0.003

* IQR, interquartile range. ** TIA, transient ischemic attack. *** COPD, chronic obstructive pulmonary disease. **** Kidney injury, defined as a glomerular filtration rate of <60 mL/min/1.73 m^2^. ***** AIDS, acquired immunodeficiency syndrome. ****** HIV, human immunodeficiency virus. ****** ICU, intensive care unit.

## Data Availability

The data sets used and/or analyzed during the current study can be made available by the corresponding author on reasonable request.

## References

[1] Flisiak R., Rzymski P., Zarębska-Michaluk D., Rogalska M., Rorat M., Czupryna P., Lorenc B., Ciechanowski P., Kozielewicz D., Piekarska A. (2022). Demographic and Clinical Overview of Hospitalized COVID-19 Patients during the First 17 Months of the Pandemic in Poland. J. Clin. Med..

[2] Current Data Polish COVID-19 Epidemiological Sutiation. https://koronawirusunas.pl/.

[3] Włodarczyk W.C. (2020). Remarks on COVID-19 Pandemic in Poland: A Health Policy Perspective. Zesz. Nauk. Ochr. Zdrowia.

[4] Ong S.W.X., Chiew C.J., Ang L.W., Mak T.M., Cui L., Toh M.P.H.S., Lim Y.D., Lee P.H., Lee T.H., Chia P.Y. (2021). Clinical and Virological Features of Severe Acute Respiratory Syndrome Coronavirus 2 (SARS-CoV-2) Variants of Concern: A Retrospective Cohort Study Comparing B.1.1.7 (Alpha), B.1.351 (Beta), and B.1.617.2 (Delta). Clin. Infect. Dis..

[5] Toyoshima Y., Nemoto K., Matsumoto S., Nakamura Y., Kiyotani K. (2020). SARS-CoV-2 genomic variations associated with mortality rate of COVID-19. J. Hum. Genet..

[6] Wrenn J.O., Pakala S.B., Vestal G., Shilts M.H., Brown H.M., Bowen S.M., Strickland B.A., Williams T., Mallal S.A., Jones I.D. (2022). COVID-19 severity from Omicron and Delta SARS-CoV-2 variants. Influenza Other Respir. Viruses.

[7] Flisiak R., Zarębska-Michaluk D., Flisiak-Jackiewicz M., Rzymski P. (2022). Updates in Management of SARS-CoV-2 Infection. J. Clin. Med..

[8] Skrzat-Klapaczyńska A., Bieńkowski C., Kowalska J., Paciorek M., Puła J., Krogulec D., Stengiel J., Pawełczyk A., Perlejewski K., Osuch S. (2022). The Beneficial Effect of the COVID-19 Vaccine Booster Dose among Healthcare Workers in an Infectious Diseases Center. Vaccines.

[9] Orlewska K., Kozieł D., Klusek J., Orlewska E. (2022). Burden of COVID-19 Mortality and Morbidity in Poland in 2020. Int. J. Environ. Res. Public Health.

[10] Flisiak R., Horban A., Jaroszewicz J., Kozielewicz D., Mastalerz-Migas A., Owczuk R., Parczewski M., Pawłowska M., Piekarska A., Simon K. (2021). Management of SARS-CoV-2 infection: Recommendations of the Polish Association of Epidemiologists and Infectiologists as of April 26, 2021. Pol. Arch. Intern. Med..

[11] Flisiak R., Parczewski M., Horban A., Jaroszewicz J., Kozielewicz D., Pawłowska M., Piekarska A., Simon K., Tomasiewicz K., Zarębska-Michaluk D. (2020). Management of SARS-CoV-2 infection: Recommendations of the Polish Association of Epidemiologists and Infectiologists. Annex no. 2 as of October 13, 2020. Pol. Arch. Intern. Med..

[12] Khodaverdi M., Price B.S., Santangelo S.L., Anzalone A., Kimble W., Porterfield J.Z., Vest M.T., Hodder S.L., Hendricks B., Rosen C.j. (2021). 447. An Ordinal Scale Assessing SARS-CoV-2 Infected Patient Outcomes Using Electronic Health Records. Open Forum Infect. Dis..

[13] von Elm E., Altman D.G., Egger M., Pocock S.J., Gøtzsche P.C., Vandenbroucke J.P. (2008). The Strengthening the Reporting of Observational Studies in Epidemiology (STROBE) statement: Guidelines for reporting observational studies. J. Clin. Epidemiol..

[14] Stidsen J.V., Green A., Rosengaard L., Højlund K. (2022). Risk of severe COVID-19 infection in persons with diabetes during the first and second waves in Denmark: A nationwide cohort study. Front. Endocrinol..

[15] Hadadi A., Pirzadeh M., Kazemian S., Ashraf H., Ebrahimi M., Karbalai Saleh S., Talebpour M. (2022). COVID-19 in Iran: Clinical presentations and outcomes in three different surges of COVID-19 infection. Virol. J..

[16] Cusinato M., Gates J., Jajbhay D., Planche T., Ong Y.E. (2022). Increased risk of death in COVID-19 hospital admissions during the second wave as compared to the first epidemic wave: A prospective, single-centre cohort study in London, UK. Infection.

[17] Bowen A., Zucker J., Shen Y., Huang S., Yan Q., Annavajhala M.K., Uhlemann A.-C., Kuhn L., Sobieszczyk M., Castor D. (2022). Reduction in Risk of Death Among Patients Admitted With COVID-19 Between the First and Second Epidemic Waves in New York City. Open Forum Infect. Dis..

[18] Ziuzia-Januszewska L., Januszewski M., Sosnowska-Nowak J., Janiszewski M., Dobrzyński P., Jakimiuk A.A., Jakimiuk A.J. (2022). COVID-19 Severity and Mortality in Two Pandemic Waves in Poland and Predictors of Poor Outcomes of SARS-CoV-2 Infection in Hospitalized Young Adults. Viruses.

[19] Oda Y., Shimada M., Shiraishi S., Kurai O. (2021). Treatment and outcome of COVID-19 patients in a specialized hospital during the third wave: Advance of age and increased mortality compared with the first/second waves. JA Clin. Rep..

[20] Fang X., Li S., Yu H., Wang P., Zhang Y., Chen Z., Li Y., Cheng L., Li W., Jia H. (2020). Epidemiological, comorbidity factors with severity and prognosis of COVID-19: A systematic review and meta-analysis. Aging.

[21] Deshmukh V., Motwani R., Kumar A., Kumari C., Raza K. (2021). Histopathological observations in COVID-19: A systematic review. J. Clin. Pathol..

[22] National Institutes of Health (2022). RT-COVAR Map of SARS-CoV-2 Variants and Mutations from National Institute of Public Health. https://covar.mi2.ai/poland/regions.

[23] Yakovleva A., Kovalenko G., Redlinger M., Liulchuk M.G., Bortz E., Zadorozhna V.I., Scherbinska A.M., Wertheim J.O., Goodfellow I., Meredith L. (2021). Tracking SARS-COV-2 Variants Using Nanopore Sequencing in Ukraine in Summer 2021. Res. Sq..

[24] GISAID Data on SARS-CoV-2 Variants in Each Country. https://covariants.org/per-country?region=World.

[25] Zhang C., Jin H., Wen Y.F., Yin G. (2021). Efficacy of COVID-19 Treatments: A Bayesian Network Meta-Analysis of Randomized Controlled Trials. Front. Public Health.

[26] (2021). Efficacy and safety of two neutralising monoclonal antibody therapies, sotrovimab and BRII-196 plus BRII-198, for adults hospitalised with COVID-19 (TICO): A randomised controlled trial. Lancet Infect. Dis..

[27] Singh B., Ryan H., Kredo T., Chaplin M., Fletcher T. (2021). Chloroquine or hydroxychloroquine for prevention and treatment of COVID-19. Cochrane Database Syst. Rev..

[28] Million M., Roussel Y., Gautret P., Raoult D. (2021). Effect of hydroxychloroquine and azithromycin on SARS-CoV-2 clearance in COVID-19 patients, a meta-analysis. Int. J. Antimicrob. Agents.

[29] Fiolet T., Guihur A., Rebeaud M.E., Mulot M., Peiffer-Smadja N., Mahamat-Saleh Y. (2021). Effect of hydroxychloroquine with or without azithromycin on the mortality of coronavirus disease 2019 (COVID-19) patients: A systematic review and meta-analysis. Clin. Microbiol. Infect..

[30] Verdejo C., Vergara-Merino L., Meza N., Pérez-Bracchiglione J., Carvajal-Juliá N., Madrid E., Rada G., Rojas Reyes M.X. (2020). Macrolides for the treatment of COVID-19: A living, systematic review. Medwave.

[31] National Institutes of Health COVID-19 Treatment Guidelines Panel. Coronavirus Disease 2019 (COVID-19) Treatment Guidelines. https://www.covid19treatmentguidelines.nih.gov.

[32] Zarębska-Michaluk D., Jaroszewicz J., Rogalska M., Martonik D., Pabjan P., Berkan-Kawińska A., Bolewska B., Oczko-Grzesik B., Kozielewicz D., Tudrujek-Zdunek M. (2021). Effectiveness of Tocilizumab with and without Dexamethasone in Patients with Severe COVID-19: A Retrospective Study. J. Inflamm. Res..

[33] Flisiak R., Jaroszewicz J., Rogalska M., Łapiński T., Berkan-Kawińska A., Bolewska B., Tudrujek-Zdunek M., Kozielewicz D., Rorat M., Leszczyński P. (2021). Tocilizumab Improves the Prognosis of COVID-19 in Patients with High IL-6. J. Clin. Med..

[34] Kowalska J.D., Skrzat-Klapaczyńska A., Bursa D., Balayan T., Begovac J., Chkhartishvili N., Gokengin D., Harxhi A., Jilich D., Jevtovic D. (2020). HIV care in times of the COVID-19 crisis—Where are we now in Central and Eastern Europe?. Int. J. Infect. Dis..

[35] Gujski M., Jankowski M., Rabczenko D., Goryński P., Juszczyk G. (2021). Characteristics and Clinical Outcomes of 116,539 Patients Hospitalized with COVID-19—Poland, March–December 2020. Viruses.

